# Neurological Comorbidity Is a Predictor of Death in Covid-19 Disease: A Cohort Study on 576 Patients

**DOI:** 10.3389/fneur.2020.00781

**Published:** 2020-07-07

**Authors:** David García-Azorín, Enrique Martínez-Pías, Javier Trigo, Isabel Hernández-Pérez, Gonzalo Valle-Peñacoba, Blanca Talavera, Paula Simón-Campo, Mercedes de Lera, Alba Chavarría-Miranda, Cristina López-Sanz, María Gutiérrez-Sánchez, Elena Martínez-Velasco, María Pedraza, Álvaro Sierra, Beatriz Gómez-Vicente, Ángel Guerrero, David Ezpeleta, María Jesús Peñarrubia, Jose Ignacio Gómez-Herreras, Elena Bustamante-Munguira, Cristina Abad-Molina, Antonio Orduña-Domingo, Guadalupe Ruiz-Martin, María Isabel Jiménez-Cuenca, Santiago Juarros, Carlos del Pozo-Vegas, Carlos Dueñas-Gutierrez, Jose María Prieto de Paula, Belén Cantón-Álvarez, Jose Manuel Vicente, Juan Francisco Arenillas

**Affiliations:** ^1^Department of Neurology, Hospital Clínico Universitario de Valladolid, Valladolid, Spain; ^2^Department of Medicine, University of Valladolid, Valladolid, Spain; ^3^Department of Neurology, Hospital Universtitario Quironsalud Madrid, Madrid, Spain; ^4^Department of Hematology, Hospital Clínico Universitario de Valladolid, Valladolid, Spain; ^5^Department of Anestesiology and Reanimation, Hospital Clínico Universitario de Valladolid, Valladolid, Spain; ^6^Department of Intensive Care Medicine, Hospital Clínico Universitario de Valladolid, Valladolid, Spain; ^7^Department of Microbiology and Immunology, Hospital Clínico Universitario de Valladolid, Valladolid, Spain; ^8^Department of Clinical Chemistry, Hospital Clínico Universitario de Valladolid, Valladolid, Spain; ^9^Department of Radiology, Hospital Clínico Universitario de Valladolid, Valladolid, Spain; ^10^Department of Pneumology, Hospital Clínico Universitario de Valladolid, Valladolid, Spain; ^11^Emergency Department, Hospital Clínico Universitario de Valladolid, Valladolid, Spain; ^12^Department of Internal Medicine, Hospital Clínico Universitario de Valladolid, Valladolid, Spain; ^13^Hospital Clínico Universitario de Valladolid, Valladolid, Spain; ^14^Neurovascular Research Laboratory, Instituto de Biología y Genética Molecular, Universidad de Valladolid – Consejo Superior de Investigaciones Científicas, Madrid, Spain

**Keywords:** SARS-CoV-2, nervous system diseases, stroke, mortality, prognosis

## Abstract

**Introduction:** Prognosis of Coronavirus disease 2019 (Covid-19) patients with vascular risk factors, and certain comorbidities is worse. The impact of chronic neurological disorders (CND) on prognosis is unclear. We evaluated if the presence of CND in Covid-19 patients is a predictor of a higher in-hospital mortality. As secondary endpoints, we analyzed the association between CND, Covid-19 severity, and laboratory abnormalities during admission.

**Methods:** Retrospective cohort study that included all the consecutive hospitalized patients with confirmed Covid-19 disease from March 8th to April 11th, 2020. The study setting was Hospital Clínico, tertiary academic hospital from Valladolid. CND was defined as those neurological conditions causing permanent disability. We assessed demography, clinical variables, Covid-19 severity, laboratory parameters and outcome. The primary endpoint was in-hospital all-cause mortality, evaluated by multivariate cox-regression log rank test. We analyzed the association between CND, covid-19 severity and laboratory abnormalities.

**Results:** We included 576 patients, 43.3% female, aged 67.2 years in mean. CND were present in 105 (18.3%) patients. Patients with CND were older, more disabled, had more vascular risk factors and comorbidities and fewer clinical symptoms of Covid-19. They presented 1.43 days earlier to the emergency department. Need of ventilation support was similar. Presence of CND was an independent predictor of death (HR 2.129, 95% CI: 1.382–3.280) but not a severer Covid-19 disease (OR: 1.75, 95% CI: 0.970–3.158). Frequency of laboratory abnormalities was similar, except for procalcitonin and INR.

**Conclusions:** The presence of CND is an independent predictor of mortality in hospitalized Covid-19 patients. That was not explained neither by a worse immune response to Covid-19 nor by differences in the level of care received by patients with CND.

## Introduction

Hypertension, diabetes, cardiovascular disorders, pulmonary disorders, and cancer have been associated with an increased risk of severe Coronavirus disease 2019 (Covid-19) and mortality (1–6). Since the first edition of the clinical management protocol of Covid-19 with severe acute respiratory infection, the World Health Organization (WHO) recommended that those patients, even if they present with mild symptoms, should be admitted to a designated unit for close monitoring (7).

The frequency, type and implications of neurological comorbidity in Covid-19 patients is largely unexplored. A recent review found a frequency ranging from 1.4 to 40%, with a pooled percentage of having a pre-existing neurological disease of 8.0% (8). The possible reasons for the heterogeneous results were the varying definitions, the lack of specific studies and underreported frequency. The impact of neurological comorbidities in Covid-19 disease is yet unknown. Covid-19 patients requiring intensive care unit (ICU) admission had prior history of cerebrovascular disease more frequently (3), but in some series, cerebrovascular disorders were classified together with cardiovascular disease (9). The aim of this study was to evaluate if the presence of comorbid chronic neurological disorders is associated with a worse prognosis in patients with Covid-19 disease.

## Methods

### Study Design

This study was done according to the registry of neurological symptoms in Covid-19 patients of the Spanish Society of Neurology and designed by the investigators. The study was done according to the strengthening the reporting in observational studies in epidemiology (STROBE) (10) statement. The study was done in the Clinic University Hospital, tertiary public hospital from Valladolid, Spain, free of charge for patients. Data were collected, analyzed and interpreted by the authors. All the authors reviewed and approved the final version of manuscript.

#### Data Sources

We collected data from the electronic medical records. The data about the history prior to the admission was collected from the admission report, emergency department (ED) history and primary care electronic records. The local authorities created a reference contact phone line, that followed patients with typical Covid-19 symptoms daily or every other day. Regarding the hospitalization period data, we gathered the medical records. Patients were treated according to the national Covid-19 management protocol standard of care (SOC) (11). The study period included all consecutive patients that were admitted to the hospital with a Covid-19 confirmed diagnosis between March 8th and April 11th, 2020. The information was reviewed from April 21st to May 1st. The source of the data was the admission department and the department of microbiology records, whilst notification of every Covid-19 positive was mandatory during the time of the study.

#### Covid-19 Disease Diagnosis

Covid-19 diagnosis was based on real-time reverse-transcriptase-polymerase-chain-reaction (RT-PCR) assay (LightMIx Modular SARS-CoV (COVID19) E-gene and LightMIx Modular SARS-CoV (COVID19) RdRP, Roche Diagnostics S.L.) of oropharyngeal-nasopharyngeal swab, sputum or lower respiratory tract sample; or was based on the presence of anti-SARS-CoV-2 IgM+IgA antibodies (COVID-19 ELISA IgM+IgA; Vircell, S.L. Granada, Spain) in serological test in patients with clinical symptoms, according to the WHO protocols (12). Patients without laboratory-confirmed diagnosis were not included. Recruitment was probabilistic and all consecutive patients were included. Only hospitalized patients were included. Data was extracted according to a predefined protocol by 13 neurologists that were involved in the treatment of Covid-19 patients. The needed time to review it patient was 20–30 min.

#### Chronic Neurological Disorders Definition

We used the WHO definition of disabling chronic neurological disorders (CND), as those neurological disorders that (a) caused persistent disability, (b) limited the individual's functioning, and (c) interfered with the person's ability to engage in activities (13). We included conditions affecting both mental and physical function. CND included dementia, movement disorders, prior stroke with long-term sequelae, neuromuscular disorders, spinal disorders, symptomatic central nervous system cancer, chronic encephalopathies or neuro-inflammatory disorders. (Full definitions in Supplementary Material). Researchers specifically assessed the presence of prior history of neurological diseases and only those conditions fulfilling (a), (b), and (c) criteria were included.

#### Variables

We analyzed demographic variables, prior medical history, clinical presentation, the course of the disease and treatment. Demographic variables included age, sex, date of symptoms onset. Regarding comorbidities, we analyzed the presence of hypertension (systemic blood pressure higher than 140/90 mmHg in two prior determinations), diabetes (fasting blood glucose >126 mg/dl on two separate tests, HbA1c > 6,5%, blood glucose level >200 mg after oral glucose overload or blood glucose level >200 mg/dl with diabetes symptoms), smoking habit (current or in the preceding 6 months), cardiovascular diseases (coronary artery disease, congenital heart diseases, cardiomyopathies, arrythmias, valvular heart disease, aortic aneurysms, and peripheral artery disease), chronic respiratory diseases (chronic obstructive pulmonary disease (COPD), asthma, occupational lung diseases, interstitial lung diseases and pulmonary hypertension), cancer (excluding epidermoid and basal cell carcinoma), immunocompromised state (congenital or acquired). We specifically evaluated the presence of CND. We analyzed baseline performance status by using the modified Ranking scale (mRS), ranging from 0 (no symptoms) to 6 (dead), being defined 3 as the presence of moderate disability and need of assistance (14).

Concerning the clinical presentation, we evaluated if the source of contagion was suspected or not, the time between the first symptom and the emergency department (ED) presentation. We analyzed the general symptoms, including fever (defined as axillary temperature equal or higher than 37.5%), asthenia, cough, cutaneous rash, dyspnea, diarrhea, chest pain, expectoration, headache, myalgia, nausea, odynophagia, rhinorrhea, and vomiting. We describe the type of Covid-19 diagnosis, either by RT-PCR or serological tests. We analyzed the frequency of abnormal chest imaging, either by X-ray or Computerized Tomography (CT). We evaluated the laboratory results on admission and the worst values during the hospitalization. The analyzed parameters were leukocytes [cell count × 10^9^ / L, reference value (RV):4–10], lymphocytes (count × 10^9^/L, RV: 0.9–5.2), platelets (count × 10^9^ /L, RV: 150–400), hemoglobin (g/dL, RV: 12–16), international normalized ratio (INR, RV: 0.8–1.3), D-dimer (ng/dL, RV: <500), lactate dehydrogenase (U/L, RV: 135–250), creatine-kinase (U/L, RV: 20–170), glomerular filtration rate corrected by body area (ml/min/1.73 m^2^, RV > 90), C-reactive protein (mg/L, RV: 1–5), procalcitonin (ng/mL, RV: < 5), interleukine-6 (pg/mL, RV < 5.9), ferritin (ng/mL, RV: 15–150). Interleukine-6 and ferritin were not available on admission. We evaluated the percentage of patients that presented abnormal results on admission and during the hospitalization. We describe the received treatment, that according to the local SOC, being the possible drug dose regimes hydroxychloroquine 400 mg bid for 5 days, lopinavir/ritonavir 400/100 mg bid, methylprednisolone 250 mg three consecutive days and interferon beta-1b (11). We also report the need of oxygen therapy, the use of mechanical ventilation, the need of Intensive Care Unit (ICU) admission and the all-cause mortality. The severity of the Covid-19 disease was defined according to the American Thoracic Society guidelines for community-acquired pneumonia (15) (Supplementary Material). We defined severe Covid-19 disease as the presence of either severe pneumonia or acute respiratory distress syndrome (ARDS) (16).

### Study Endpoints

The primary endpoint was in-hospital mortality of Covid-19 in patients with CND, calculated by multivariate regression and survival probability by Cox regression, adjusted by the possible confounders and effect modifiers. As secondary endpoints, we aimed to analyze the Covid-19 severity in CND patients and the presence of Covid-19 related laboratory abnormalities.

### Statistical Analysis

Qualitative and ordinal variables are expressed as frequency and percentage. Continuous variables are presented as medians, interquartile range (IQR), and minimum-maximum value or mean and standard deviation (SD). Missing data was managed by complete case analysis. In the statistical analysis we employed Chi^2^ test or Fisher's Exact test for the contrast of categorical variables, adjusting *p*-value by Bonferroni method for multiple comparisons correction. We employed Student *T*-test for the contrast of categorical and continuous variables. The level of significance threshold was set in 0.05, after adequate adjustment. Due to the exploratory nature of the study, we did not calculate sample size.

For the primary endpoint, we conducted a univariate regression analysis of all baseline variables and all the variables that showed statistical association with higher odds of death and a *p*-value lower or equal than 0.1, were included in a multivariate regression analysis. All CND were analyzed together. We present the odds ratio (OR) and the 95% confidence interval (CI). Survival probability over time was assessed by Cox-regression analysis with hazard ratio (HR) and the corresponding 95% CI analysis, adjusted by all the covariates that were significant in the univariate regression analysis. Differences in Kaplan Meier Curves were analyzed by the log-rank test.

For the secondary endpoint severe-Covid-19 disease, we repeated the same analysis as for the primary endpoint. To evaluate if laboratory parameters were more often abnormal in patients with CND, we created a regression analysis adjusting for age, mRS and sex. Statistical analysis was performed with SPSS v.26 (IBM Corp. Armonk, NY) by DGA. All datasheets are available for other researchers under reasonable request.

### Ethical Aspects

The study protocol was approved by the institutional review board of Valladolid Este health area (PI-20-1751). Written informed consent was waived given the risk of contagion and the urgent need of data.

### Data Availability

Datasheets are available for other researchers under reasonable request.

## Results

During the study period, 580 consecutive patients were admitted and hospitalized to our hospital with a positive test for SARS-CoV-2, being excluded four of them. Supplementary Figure 1 shows the flow diagram of patients. The sample included thus 576 patients, 250 (43.3%) female, with a mean age of 67.2 (sd:14.7), ranging from 23 to 98 years. One hundred and five (18.3%) patients had one or more chronic neurological disorders (CND). The frequency of specific disorders was 40 (6.9%) patients with cerebrovascular disease, 32 (5.5%) patients with cognitive disorders, 24 (4.1%) patients with neuromuscular and spinal diseases, 16 (2.7%) patients with movement disorders, four (0.7%) patients with symptomatic central nervous system tumors, two (0.3%) patients with multiple sclerosis and isolated cases of Neurobehçet disease, neurolupus and one malformation syndrome. The full list of patients is available in Supplementary Materials. Patients with CND were older, more disabled at baseline and had hypertension and diabetes more often. Table 1 shows demographic variables, vascular risk factors frequency and comorbidities.

**Table 1:** Demographic variables, vascular risk factors frequency and comorbidities.

	**All patients (*n* = 576)**	**Chronic neurological disorders (*n* = 105)**	**No-neurological comorbidity (*n* = 471)**	**Adjusted *p*-value**
Mean age	67.18 (14.75)	74.97 (12.69)	65.45 (14.63)	<0.001^†^
Female sex	250 (43.4%	50 (47.6%)	200 (42.5%)	0.384^‡^
Hypertension	300 (52.1%)	75 (71.4%)	225 (47.8%)	<0.001^‡^
Diabetes	113 (19.6%)	34 (32.4%)	79 (16.8%)	0.001^‡^
Smoking habit	118 (20.5%)	22 (21.0%)	96 (20.4%)	0.894^‡^
Cardiac disease	154 (26.7%)	41 (39.0%)	113 (24.0%)	0.002^‡^
Respiratory disease	145 (25.2%)	31 (29.5%)	114 (24.2%)	0.264^‡^
Cancer	94 (16.3%)	22 (21.0%)	72 (15.3%)	0.188^‡^
Immunodepression	32 (5.6%)	10 (0.5%)	22 (4.7%)	0.059^‡^
Mean mRS	0.61 (1.12)	1.73 (1.48)	0.36 (0.84)	<0.001^†^

*mRS, Modified Rankin Scale*;

† *Student T-Test*;

‡ *Two-sided Fisher's Exact test*.

The source of the contagion was suspected in 282 (49%) of all cases, without differences between patients with CND (58 (55.2%) cases) and the rest of the patients (224 (47.6%) cases). The most frequent symptoms on presentation were fever and cough. Patients with CND had less frequently cough, asthenia, diarrhea, myalgia, chest pain, headache or lightheadedness. The frequency and type of general presenting symptoms is shown in Supplementary Table 3.

### Latency Between Symptom Onset and ED Presentation

**Figure 1:**
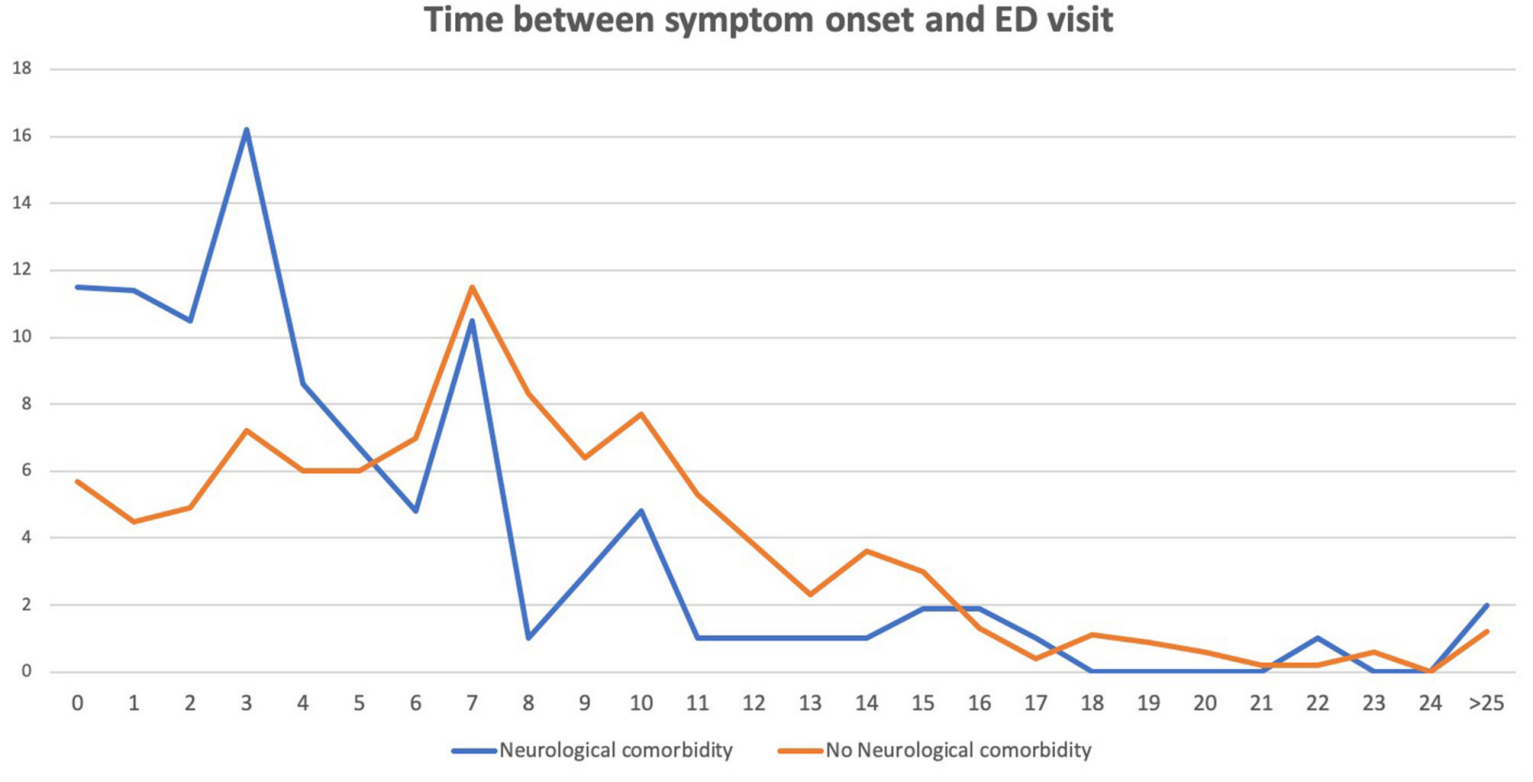
Time between the first symptom and the emergency department (ED) visit in days. *Y axis: Percentage of patients. X axis: Days since the first symptom to the ED presentation*.

The mean time between symptom onset and ED visit in patients with neurological comorbidities was 5.27 (sd: 7.72) days, compared with 7.81 (sd: 5.66) days in those without prior neurological history. After adjusting for age, mRS, sex, vascular risk factors and comorbidities, linear regression analysis was significant (B coefficient −1.436, 95% CI: −2.844-−0.28, *p* = 0.046). Figure 1 shows the interval between symptom onset and ED visit in patients with and without neurological comorbidities in patients with and without CND.

### Diagnosis and Management of Patients

Diagnosis was confirmed by RT-PCR in 546 (94.8%) of cases and/or serology in 175 (30.4%). Chest imaging was abnormal in 549 patients (95.3%). Patients with CND received hydroxychloroquine (83.8 vs. 92.6%, *p* = 0.008) and lopinavir/ritonavir (81.9 vs. 92.1%, *p* = 0.003) less frequently. Frequency of methylprednisolone or interferon use was similar. Need of oxygen therapy was more frequent in CND patients (83.8 vs. 66.2%, *p* = 0.001). Frequency of ventilatory support or ICU admission was similar.

### Course of the Disease

Concerning the clinical course, 393 (68.2%) of patients had a severe pneumonia or ADRS and 127 (22.0%) died. Nine patients had not pneumonia but had severe illness because of septic shock 5 (0.8%), pulmonary embolism without pneumonia 2 (0.3%), and one case (0.1%) of lithium intoxication and one case (0.1%) of fatal gastrointestinal bleed. Patients with CND had non-severe pneumonia less frequently (12.4 vs. 27.4%, *p* = 0.002) and ADRS more frequently (30.5 vs. 19.6%, *p* = 0.020). Mortality of CNS patients was 44.8%, compared with 17% in the rest of the sample (*p* < 0.001). Supplementary Table 4 describes treatment and severity of Covid-19 disease.

### Primary Endpoint: Predictors of Mortality

In the univariate regression analysis, baseline disability, age, hypertension, diabetes, smoking habit, cardiac disorders, cancer and chronic neurological disorders were associated with higher odds of mortality, whereas female sex was associated with a lower odd of death. In the multivariate regression analysis, including all the variables that were statistically significant in the univariate analysis, baseline disability, age and chronic neurological disorders remained statistically significant (OR: 1.76, 95% CI: 1.014–3.06). Table 2 presents the results of the univariate and multivariate regression analysis.

**Table 2:** Predictors of mortality: univariate and multivariate regression analysis.

	**Type of analysis**	**OR**	**95% CI**	***p*-value**
mRS≥3	Univariate	11.371	6.376–20.278	<0.001
	Multivariate	4.100	2.088–8.050	<0.001
Age	Univariate	1.090	1.069–1.112	<0.001
	Multivariate	1.064	1.040–1.089	<0.001
Female sex	Univariate	0.682	0.454–1.024	0.065
	Multivariate	0.770	0373–1.052	0.077
Hypertension	Univariate	3.534	2.272–5.495	<0.001
	Multivariate	1.369	0.806–2.325	0.246
Diabetes	Univariate	2.129	1.353–3.351	0.001
	Multivariate	1.221	0.710–2.098	0.471
Smoking	Univariate	1.589	1.004–2.514	0.048
	Multivariate	1.720	0.955–3.096	0.701
Cardiological disorders	Univariate	2.955	1.950–4.478	<0.001
	Multivariate	1.208	0.730-1.999	0.462
Pulmonary disorders	Univariate	1.434	0.928–2.217	0.105
	Multivariate	0.931	0.543–1.596	0.794
Cancer	Univariate	1.641	1.001–2.690	0.049
	Multivariate	1.209	0.676–2.162	0.523
Chronic neurological disorders	Univariate	3.961	2.516–6.234	<0.001
	Multivariate	1.763	1.014–3.064	0.044
Immunosuppression	Univariate	1.295	0.405–4.138	0.663

*mRS, Modified Rankin Scale; OR, Odds Ratio; CI, Confidence Interval*.

**Figure 2:**
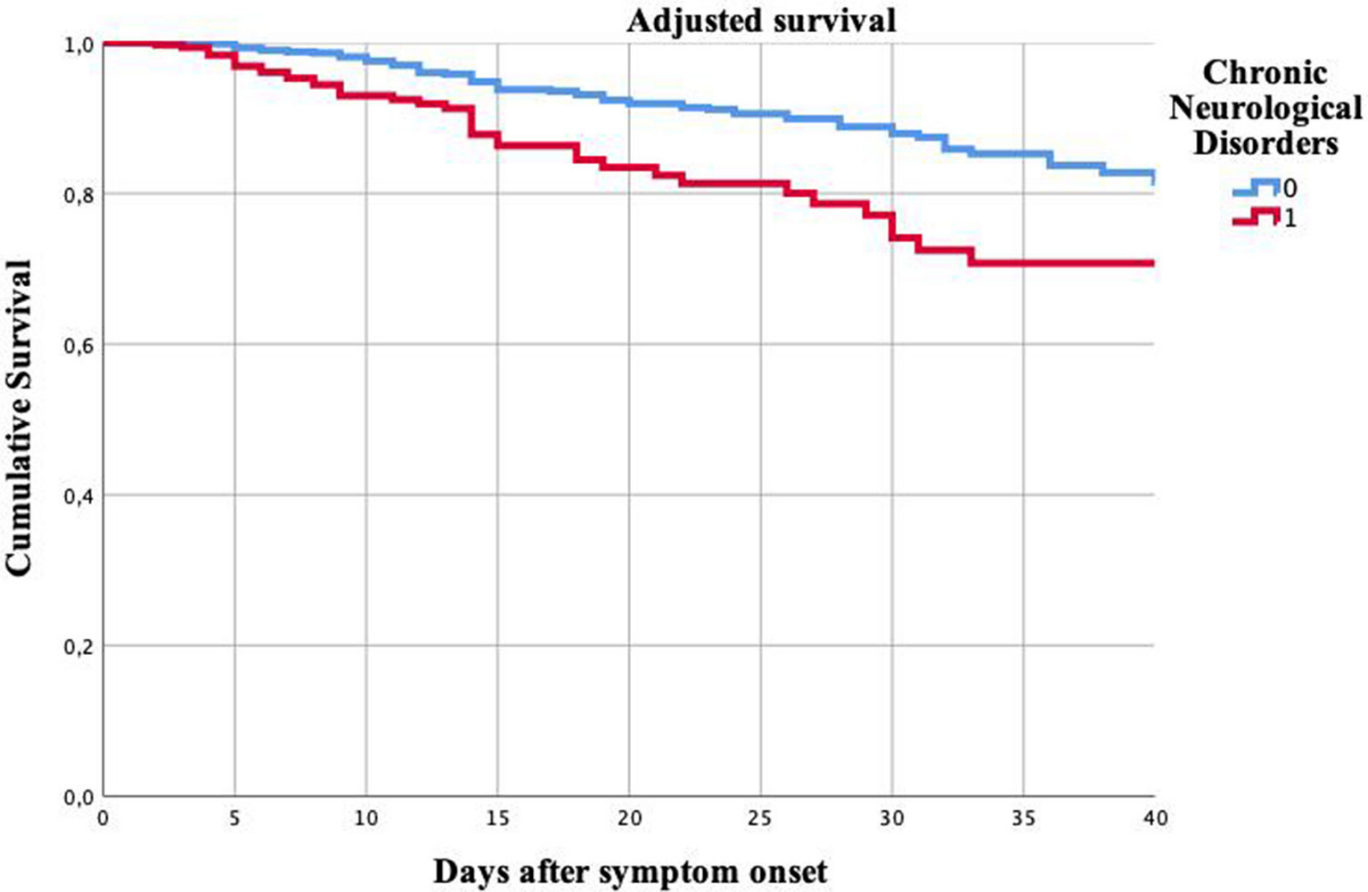
Cumulative survival of patients with and without chronic neurological disorders. Kaplan Meier curves. *Y axis: Cumulative survival. X axis: Days after the symptoms onset*.

Cox regression analysis patients with CND had lower survival over time than patients without prior history of CND (HR: 2.13, 95% CI: 1.382–3.280, *p* = 0.001), adjusted by all the variables included in the multivariate regression analysis (age, mRS, sex, presence of hypertension, diabetes, smoking habit, prior history of cardiac disorders, pulmonary diseases, and history of cancer). Figure 2 shows cumulative survival curves. Supplementary Table 5 presents the results of all the variables included in the analysis.

### Predictors of Severe Covid-19 Disease

All the variables that were associated with higher odds of mortality except by cancer were associated with a higher odd of severe Covid-19 disease in the univariate regression analysis. In the multivariate analysis, only age, female sex and diabetes remained statistically significant, with a trend to signification in smoking habit (*p* = 0.066) and CND (*p* = 0.063). Table 3 shows results of univariate and multivariate regression analysis.

**Table 3:** Predictors of severe Covid-19 disease. Univariate and multivariate regression analysis.

	**Type of analysis**	**OR**	**95% CI**	***p*-value**
mRS>2	Univariate	2.437	1.241–4.786	0.010
	Multivariate	1.272	0.587–2.756	0.542
Age	Univariate	1.031	1.019–1.044	<0.001
	Multivariate	1.029	1.013–1.045	<0.001
Female sex	Univariate	0.584	0.410–0.832	0.003
	Multivariate	0.642	0.437–0.942	0.024
Hypertension	Univariate	1.485	1.044–2.113	0.028
	Multivariate	0.802	0.522–1.232	0.314
Diabetes	Univariate	2.714	1.600–4.603	<0.001
	Multivariate	2.200	1.256–3.855	0.006
Smoking	Univariate	2.083	1.279–3.392	0.003
	Multivariate	1.648	0.967–2.809	0.066
Cardiological disorders	Univariate	1.527	1.008–2.314	0.046
	Multivariate	0.834	0.516–1.350	0.460
Pulmonary disorders	Univariate	1.812	1.172-2.801	0.008
	Multivariate	1.399	0.874–2.240	0.162
Cancer	Univariate	1.534	0.924-2.547	0.098
	Multivariate	1.182	0.689–2.027	0.523
Chronic neurological disorders	Univariate	2.418	1.421–4.115	0.001
	Multivariate	1.750	0.970–3.158	0.063
Immunosuppression	Univariate	1.289	0.405–4.103	0.668

*ADRS, Acute Distress Respiratory Syndrome; mRS, Modified Rankin Scale; OR, Odds Ratio; CI, Confidence Interval*.

### Laboratory Findings

The median laboratory values on admission and the worst values during hospitalization is available in Supplementary Table 6. All the laboratory parameters were more frequently abnormal during the hospitalization than upon admission (all *p* < 0.001), Supplementary Table 7. Patients with CND had higher odds of having increased INR during hospitalization (OR: 1.85, 95% CI: 1.14–3.01) and higher odds of having increased procalcitonin levels during hospitalization (OR: 1.845, 95% CI: 1.08–3.15), after adjusting for age, mRS, sex, and prior history of hypertension, diabetes, smoking habit and other comorbidities. Supplementary Table 8 shows the full results of the regression analysis.

## Discussion

Most of the Covid-19 management protocols coincide that patients with comorbidities should be closely monitored (7, 11), however specific recommendations for neurological comorbidities are sparse. Frequency of CND comorbidities in Covid-19 patients have not been considered in most of the studies (8). To our knowledge, this is the first study that analyzes the implication of CND presence. CND was an independent predictor of in-hospital mortality, and death occurred faster in CND patients.

As expected, patients with CND were older, more disabled and had higher frequency of vascular risk factors and other comorbidities. All of those have been associated with higher mortality in most of the Covid-19 series (1–6). The clinical presentation of Covid-19 disease was pauci symptomatic in CND patients, as some typical symptoms such as cough, chest pain or asthenia were less frequent. Also, the most frequently reported neurological symptoms, myalgia, and headache (17–19), were less common. Despite of that, CND patients experienced ADRS more often and had a higher death rate.

Considering the more severe disease, the worst baseline performance and the scanty clinical expression, the reason for the different outcome could be related with a delayed ED presentation. However, in our sample, patients with CND came earlier, even after adjusting for age, mRs, sex, and the rest of comorbidities. Hence, we could not attribute the worse prognosis to the delay in the care provision (6).

Then, we tried to find out if patients with CND received standard care and/or intensive care less often. More than 80% of CND patients received pharmacological treatment according to the local SOC. Even though the clinical benefit of those drugs is not yet clear (20), we could not attribute the worse prognosis to the restriction of the received therapy. Due to the collapse of the sanitary system, ICU guidelines deemed to prioritize the resource allocation to those patients with a higher potential benefit (21). Nevertheless, in our sample, patients with CND benefited from ICU admission as frequently as those without it.

Since the need of oxygen therapy was more frequent in patients with CND, a possible explanation of the higher mortality was the more severe Covid-19 disease. As the simple comparison of mean or median values of laboratory findings was not adequate due to the imbalanced populations, we analyzed if patients with CND had abnormal laboratory values more frequently than the rest of the patients, adjusting by all the variables that deemed potential confounders. Only increased INR and procalcitonin remained statistically significant. The reason why INR is more frequently increased in patients with CND is elusive and could be related with hepatic failure caused by sepsis, low K vitamin levels, or acute liver failure (22). In the case of procalcitonin, patients with CND are more vulnerable to nosocomial infections (23), so the bacterial co-infection could be the most likely explanation (24, 25). It has been pointed as an independent predictor of fatal outcome in Covid-19 patients (26).

Henceforth, if we could not attribute the worse prognosis of CND patients neither to a delayed presentation to the ED nor a different management (27), a plausible explanation could be the higher fragility and lower reserve of CND population. It is well-known that CND are independent predictors of mortality in hospitalized patients (28, 29). Prior history of stroke has been related with a higher odds of severe Covid-19 illness, as well (30). The possible reasons seem varied, including frequency of delirium (18, 19), malnutrition (31), impaired respiratory function (32), and worse self-management (33). Many of them can be worsened by Covid-19 disease and the use of personal protective equipment makes its management arduous. Covid-19 should be prevented and detected early in CND patients, whose close monitoring could prevent complications and improve the prognosis (34). In addition, the worse prognosis of patients with CND could be linked with immuno-senescence, an enhanced inflammatory state, favored by an angiotensin II induced vasoconstriction and inflammatory response, leading to lymphopenia, cytokine release and macrophage activation (35).

This study has notable limitations. First, the sample size was modest, implying the possibility of some false-negative results. The number of CND was not high enough to perform sub-studies by different specific conditions, but all the patients were consecutive. We tried to create an operative definition of CND, based on the persistent impact on functionality, however the definition is imperfect and not every neurological disorder might be equally relevant. Further studies should focus on the specific impact of the different neurological comorbidities and analyze them separately. Second, this was a single center study, it would be desirable to create multicentric studies to clarify the impact of other variables, such as the type of hospital care and the different management of patients. Future studies should be multinational, as the reported adjusted mortality rates are highly variable between the different countries. It is not yet known if it could be attributed to genetic predisposition for severe Covid-19 or different healthcare systems. This was a retrospective study and despite the information was carefully reviewed, some information could be incomplete. It was a limitation that there were many different researchers involved in the study, albeit all of them were neurologists and followed a pre-defined protocol. We did not include long-term follow up of the patients, some patients remained admitted at the time of data cutoff, mortality might be underestimated in some cases. Also, the sample is not representative of the whole population, as it only included hospitalized patients, which could influence the results.

## Conclusion

The presence of pre-existing chronic neurological disorders was an independent predictor of mortality in hospitalized Covid-19 patients.

Death occurred faster after admission in patients with CND, and CND was associated with an earlier presentation at ED. Presence of CND was not associated with a worse inflammatory response or with differences in the level of care provided to the patients.

The course of Covid-19 in patients with CND appears to be faster and more aggressive, and therefore protocols should consider these patients as a very high-risk population.

Future Covid-19 studies should consider the presence of CND in the evaluation of risk of mortality.

## Data Availability Statement

The raw data supporting the conclusions of this article will be made available by the authors, without undue reservation. Datasheets are available upon reasonable request to the corresponding author.

## Ethics Statement

The studies involving human participants were reviewed and approved by Valladolid Este Ethics Review Board. The ethics committee waived the requirement of written informed consent for participation.

## Author Contributions

DG-A and JA designed and conceptualized the study. DG-A, EM-P, JT, IH-P, GV-P, BT, PS-C, ML, AC-M, CL-S, MG-S, EM-V, and MP collected the data. DG-A and JA analyzed and interpreted the data. DG-A drafted the manuscript. ÁS, BG-V, ÁG, DE, MJP, JG-H, EB-M, CA-M, AO-D, GR-M, MJ-C, SJ, CP-V, CD-G, JP, BC-Á, and JV revised the manuscript for intellectual content. All authors agreed and approved the publication.

## Conflict of Interest

The authors declare that the research was conducted in the absence of any commercial or financial relationships that could be construed as a potential conflict of interest. The handling editor declared a past co-authorship with several of the authors DG-A, MG-S.
