# Supplementary material for: Neurological Comorbidity Is a Predictor of Death in Covid-19 Disease: A Cohort Study on 576 Patients
Source: Front Neurol. 2020 Jul 7;11:781. doi: 10.3389/fneur.2020.00781 (PMC7358573; doi:10.3389/fneur.2020.00781)
Supplement: Supplementary file 1 [file Table_1.DOCX]

Supplementary Material

Supplementary table 1:

Chronic neurological disorders:

Neurological disorders fulfilling criteria A-C:

A: Causing persistent disability.

B: Limiting individuals’ functioning.

C: Affecting person’s ability to engage in activities.

| Dementia | Alcoholic dementia, Alzheimer disease, Vascular dementia, Frontotemporal dementia, Creutzfeldt Jacob Disease, Hepatolenticular degeneration, Dementia associated with chronic infections, dementia in patients with movement disorders, dementia associated with B12 vitamin deficiency, normal pressure hydrocephalus. |
| --- | --- |
| Movement disorders | Parkinson disease, Progressive Supranuclear Palsy, dementia with Lewy body, Corticobasal syndrome, ataxia syndromes, Huntington chorea, Wilsons’s disease, dystonic syndromes. |
| Prior stroke | Causing long-term physical or cognitive sequelae. |
| Neuromuscular disorders | Neuromuscular junction disorders, myopathies, neuropathies, radiculopathies, plexopathies. |
| Spinal disorders | Cervical, thoracic or lumbar spinal stenosis; myelopathies. |
| Symptomatic central nervous system cancer | Primary central nervous system tumors, metastases, meningeal carcinomatosis, paraneoplastic syndromes. |
| Chronic encephalopathies | Hypoxic encephalopathy, chronic metabolic encephalopathy, Wernicke encephalopathy. |
| Neuro-inflammatory diseases | Multiple sclerosis with long-term sequelae, systemic vasculitis, Lupus, Behçet, Sjögren, sarcoidosis. |

Severity of Covid-19 disease according to the American Thoracic Society guidelines for community-acquired pneumonia^15^.

| Severity | Description |
| --- | --- |
| Mild illness | Patients with uncomplicated upper respiratory tract viral infection symptoms and have non-specific symptoms such as fever, fatigue, cough (with or without sputum production), anorexia, malaise, muscle pain, sore throat, dyspnea, nasal congestion, diarrhea, nausea or vomiting or |
| Pneumonia | Presence of pneumonia but no signs of severe pneumonia and no need for supplemental oxygen.  CURB scale≤1. |
| Severe pneumonia | Confirmed respiratory infection, plus one of the following:   1. Respiratory rate > 30 breaths/min. 2. Severe respiratory distress. 3. SpO2 ≤ 93% on room air. |
| Acute respiratory distress syndrome (ARDS)^16^ | **Onset:** within 1 week of a known clinical insult or new or worsening respiratory symptoms.  **Chest imaging** (radiograph, CT scan, or lung ultrasound): bilateral opacities, not fully explained by volume overload, lobar or lung collapse, or nodules.  **Origin of pulmonary infiltrates**: respiratory failure not fully explained by cardiac failure or fluid overload. Need objective assessment (e.g. echocardiography) to exclude hydrostatic cause of infiltrates/oedema if no risk factor present. **Oxygenation impairment in adults**:   - Mild ARDS: 200 mmHg < PaO2/FiO2a ≤ 300 mmHg (with PEEP or CPAP ≥ 5 cmH2O, or non-ventilated) - Moderate ARDS: 100 mmHg < PaO2/FiO2 ≤ 200 mmHg (with PEEP ≥ 5 cmH2O, or non-ventilated) - Severe ARDS: PaO2/FiO2 ≤ 100 mmHg (with PEEP ≥ 5 cmH2O, or non-ventilated) - When PaO2 is not available, SpO2/FiO2 ≤ 315 implies ARDS (including in non-ventilated patients). |

*Sp: Saturation percentage. ADRS: Acute Distress Respiratory Syndrome. CT: Cranial Tomography. PaO2: Partial pressure of Oxygen. FiO2: Fraction of inspired Oxygen. PEEP: Positive end-expiratory pressure. CPAP: Continuous positive airway pressure.*

Supplementary figure 1: Flow diagram of screened and included patients.


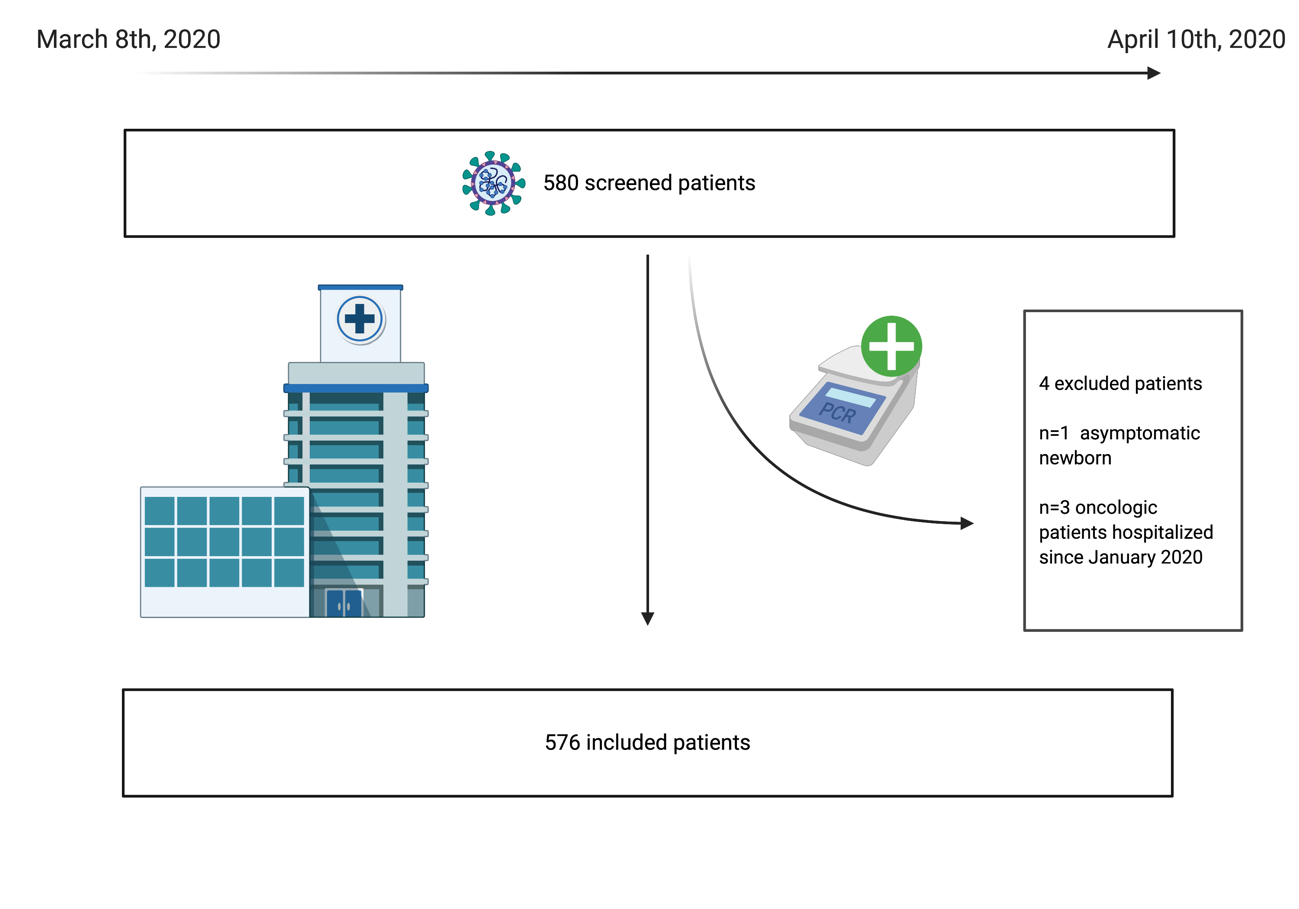


Supplementary table 2:

Full list of diagnosis, including sex and age of patients.

Note: In some patients more than one neurological disorder is listed, but not all the listed disorders were considered as CND (e.g.: Migraine, transient global amnesia, essential tremor).

| **Sex** | **Age** | **Diagnosis** |
| --- | --- | --- |
| Male | 82 | Ischemic stroke and Alzheimer |
| Female | 92 | Ischemic stroke and Alzheimer |
| Female | 81 | Ischemic stroke and Alzheimer |
| Male | 89 | Ischemic stroke and Alzheimer |
| Female | 72 | Stroke, ataxia, transient global amnesia |
| Female | 28 | Hemorrhagic stroke, dystonia, migraine |
| Female | 75 | Ischemic stroke, essential tremor, restlessness legs syndrome |
| Male | 79 | Ischemic stroke, spinal stenosis |
| Male | 70 | Ischemic stroke, radiculopathy |
| Male | 85 | Ischemic stroke, spinal stenosis |
| Female | 74 | Ischemic stroke, symptomatic epilepsy |
| Male | 86 | Hemorrhagic stroke, epilepsy |
| Female | 64 | Hemorrhagic stroke |
| Male | 72 | Ischemic stroke, subclavian steal syndrome, headache |
| Male | 66 | Ischemic stroke |
| Female | 60 | Ischemic stroke |
| Male | 72 | Ischemic stroke, meningioma |
| Female | 54 | Ischemic stroke |
| Male | 59 | Ischemic stroke |
| Male | 72 | Ischemic stroke |
| Female | 76 | Ischemic stroke |
| Male | 76 | Ischemic stroke |
| Female | 79 | Ischemic stroke |
| Female | 91 | Ischemic stroke |
| Male | 77 | Ischemic stroke |
| Female | 91 | Ischemic stroke |
| Female | 88 | Ischemic stroke with hemorrhagic transformation |
| Female | 44 | Ischemic stroke |
| Male | 75 | Ischemic stroke |
| Female | 67 | Ischemic stroke |
| Male | 78 | Ischemic stroke |
| Male | 76 | Ischemic stroke |
| Male | 72 | Ischemic stroke, vertigo |
| Male | 76 | Ischemic stroke |
| Male | 78 | Ischemic stroke |
| Female | 77 | Ischemic stroke |
| Male | 75 | Ischemic stroke |
| Male | 63 | Hemorrhagic stroke |
| Male | 89 | Ischemic stroke |
| Male | 83 | Ischemic stroke |
| Male | 77 | Parkinson disease with cognitive impairment |
| Male | 84 | Parkinson disease with cognitive impairment |
| Female | 93 | Parkinson disease, Alzheimer disease |
| Male | 80 | Dementia with Lewy body |
| Female | 89 | Alzheimer disease, myelopathy |
| Female | 87 | Alzheimer disease, epilepsy |
| Female | 62 | Alzheimer disease |
| Female | 88 | Alzheimer disease |
| Male | 77 | Alzheimer disease |
| Female | 87 | Alzheimer disease |
| Male | 87 | Alzheimer disease |
| Male | 72 | Normal pressure hydrocephalus, spinal stenosis |
| Female | 94 | Alzheimer disease |
| Female | 86 | Alzheimer disease |
| Male | 76 | Multifactorial cognitive impairment |
| Female | 83 | Alzheimer disease |
| Female | 87 | Alzheimer disease |
| Male | 87 | Alzheimer disease |
| Male | 84 | Alzheimer disease |
| Female | 87 | Alzheimer disease |
| Female | 89 | Alzheimer disease |
| Female | 83 | Alzheimer disease |
| Female | 77 | Alzheimer disease |
| Male | 94 | Alzheimer disease |
| Male | 86 | Alzheimer disease |
| Female | 97 | Alzheimer disease |
| Female | 73 | Frontotemporal dementia |
| Male | 83 | Alzheimer disease |
| Male | 64 | Parkinson disease |
| Female | 84 | Ataxia, neurolupus |
| Male | 77 | Parkinson disease |
| Male | 83 | Parkinson disease |
| Female | 73 | Acquired ataxia (possible multiple system atrophy) |
| Female | 75 | Acquired ataxia |
| Male | 65 | Parkinson disease |
| Male | 89 | Parkinson disease |
| Male | 83 | Parkinson disease |
| Female | 56 | Parkinson disease |
| Female | 74 | Hydrocephalus, Type II Chiari syndrome |
| Male | 48 | Hydrocephalus, Type II Chiari syndrome |
| Female | 60 | Polyneuropathy |
| Female | 74 | Severe spinal stenosis |
| Male | 65 | Myopathy |
| Female | 74 | Polyneuropathy, spinal stenosis |
| Male | 77 | Polyneuropathy, neuropathic tremor |
| Male | 50 | Spina bifida and spinal stenosis |
| Female | 68 | Severe spinal stenosis |
| Male | 70 | Spinal stenosis |
| Male | 70 | Myasthenia gravis |
| Male | 64 | Severe spinal stenosis |
| Male | 80 | Severe spinal stenosis |
| Male | 75 | Severe spinal stenosis, subjective memory complaints |
| Male | 65 | Polyneuropathy |
| Male | 73 | Polyneuropathy, myelopathy, spinal stenosis |
| Female | 59 | Polyneuropathy |
| Female | 78 | Polyneuropathy |
| Female | 79 | Multiple sclerosis |
| Male | 54 | Multiple sclerosis |
| Female | 78 | Alzheimer disease |
| Female | 65 | Myelopathy, antiphospholipid syndrome |
| Male | 26 | Malformation syndrome |
| Female | 57 | Neurobehçet |
| Female | 76 | Ataxia |
| Female | 73 | Brain metastases |
| Male | 79 | Multiform glioblastoma |

Supplementary table 3: *Frequency and type of general presenting symptoms in the whole sample and the two groups:*

| Symptom | All patients  (n=576) | Chronic neurological disorders (n=105) | No-neurological comorbidity  (n=471) | Adjusted p-value |
| --- | --- | --- | --- | --- |
| Fever | 462 (80.2%) | 83 (79.0%) | 379 (80.6%) | 0.814 |
| Cough | 403 (69.9%) | 61 (58.1%) | 342 (72.9%) | 0.004 |
| Dyspnea | 292 (50.7%) | 54 (51.4%) | 238 (50.6%) | 0.969 |
| Asthenia | 242 (42.0%) | 33 (31.4%) | 209 (44.5%) | 0.019 |
| Diarrhea | 192 (33.3%) | 25 (23.8%) | 167 (35.5%) | 0.029 |
| Headache | 133 (23.1%) | 10 (9.5%) | 123 (26.2%) | <0.001 |
| Myalgia | 129 (22.4%) | 15 (14.3%) | 114 (24.3%) | 0.037 |
| Chest pain | 99 (17.2%) | 10 (9.6%) | 89 (18.9%) | 0.033 |
| Weakness | 90 (15.6%) | 15 (14.3%) | 75 (16%) | 0.813 |
| Expectoration | 90 (15.7%) | 16 (15.2%) | 74 (15.7%) | 1.000 |
| Odynophagia | 60 (10.4%) | 6 (5.7%) | 54 (11.5%) | 0.116 |
| Lightheadedness | 56 (9.7%) | 3 (2.9%) | 53 (11.3%) | 0.014 |
| Vomiting | 47 (8.2%) | 11 (10.5%) | 36 (7.7%) | 0.450 |
| Arthralgia | 35 (6.1%) | 6 (5.7%) | 29 (6.2%) | 1.000 |
| Rhinorrhea | 12 (2.1%) | 2 (1.9%) | 10 (2.1%) | 1.000 |
| Rash | 11 (1.9%) | 4 (3.8%) | 7 (1.5%) | 0.240 |

*P-value adjusted for multiple comparisons.*

Supplementary table 4: Treatment received per group and severity of Covid-19 disease:

| Variable | All patients  (n=576) | Chronic neurological disorders (n=105) | No-neurological comorbidity  (n=471) | Adjusted p-value |
| --- | --- | --- | --- | --- |
| Lopinavir/ritonavir | 520 (90.3%) | 86 (81.9%) | 434 (92.1%) | 0.003 |
| Hydroxychloroquine | 524 (91.0%) | 88 (83.8%) | 436 (92.6%) | 0.008 |
| Methylprednisolone | 300 (52.3%) | 60 (57.1%) | 240 (51.2%) | 0.318 |
| Interferon Beta | 223 (38.7%) | 48 (45.7%) | 175 (37.2%) | 0.129 |
| Oxygen therapy | 400 (69.4%) | 88 (83.8%) | 312 (66.2%) | 0.001 |
| Any Ventilatory support | 101 (17.5%) | 23 (21.9%) | 78 (16.6%) | 0.246 |
| Invasive ventilation | 82 (14.2%) | 16 (15.2%) | 66 (14.0%) | 0.865 |
| ICU admission | 84 (14.6%) | 16 (15.2%) | 68 (14.4%) | 0.954 |
| Mild disease | 32 (5.6%) | 3 (2.9%) | 29 (6.2%) | 0.272 |
| Pneumonia | 142 (24.7%) | 13 (12.4%) | 129 (27.4%) | 0.002 |
| Severe pneumonia | 269 (46.8%) | 54 (51.4%) | 215 (45.7%) | 0.344 |
| ADRS | 124 (21.6%) | 32 (30.5%) | 92 (19.6%) | 0.020 |
| Death | 127 (22.0%) | 47 (44.8%) | 80 (17.0%) | <0.001 |

*ICU: Intensive Care Unit. ADRS: Acute Distress Respiratory Syndrome. P-value adjusted for multiple comparisons.*

Supplementary table 5: Predictors of mortality. Cox-regression multivariate analysis:

|  | HR | 95% CI | p-value |
| --- | --- | --- | --- |
| mRS>2 | 2.725 | 1.695-4.380 | <0.001 |
| Age | 1.057 | 1.034-1.080 | <0.001 |
| Female sex | 0.844 | 0.543-1.311 | 0.450 |
| Hypertension | 1.426 | 0.880-2.313 | 0.150 |
| Diabetes | 1.217 | 0.790-1.874 | 0.373 |
| Smoking | 1.355 | 0.833-2.204 | 0.221 |
| Cardiological disorders | 0.969 | 0.635-1.480 | 0.885 |
| Pulmonary disorders | 0.846 | 0.540-1.324 | 0.463 |
| Cancer | 0.881 | 0.537-1.446 | 0.617 |
| Chronic neurological disorders | 2.129 | 1.382-3.280 | 0.001 |

*HR: Hazard Ratio. CI: Confidence Interval. mRS: modified Rankin Scale.*

Supplementary table 6. Median values of laboratory findings on admission and the worst values during the hospitalization period.

|  | **Median value on admission** | **Median worst value** |
| --- | --- | --- |
| Leukocytes  (RV 4-10)  Units: count x 10^9^/L | 6710  (IQR: 4945-8885)  Min: 590  Max: 221220 | 9440  (IQR: 4440 – 14210)  Min: 240  Max: 389530 |
| Lymphocytes (RV 0.9-5.2)  Units: count x 10^9^/L | 990  (IQR: 710-1387.5)  Min: 80  Max: 213110 | 690  (IQR: 430 – 1050)  Min: 50  Max: 82530 |
| Hemoglobin  (RV 12-16)  Units: count x 10^9^/L | 13.5  (IQR: 12.2-14.7)  Min: 5.8  Max: 20,3 | 12  (IQR: 10.1 – 13.3)  Min: 5  Max: 18.3 |
| Platelets  (RV 150-400)  Units: count x 10^9^/L | 194  (IQR: 156-255)  Min: 15000  Max: 611000 | 192.5  (IQR: 137 – 341.2)  Min: 5  Max: 774 |
| LDH  (RV 135-250)  Units: U/L | 284  (IQR: 224.2-371.7)  Min: 58  Max: 1255 | 341  (IQR: 259 – 462)  Min: 125  Max: 35554 |
| Glomerular filtration rate (corrected by body are)  (RV >90)  Units: ml/min/1.73m^3^ | 79  (IQR: 56-90)  Min: 1  Max: >90 | 70  (IQR: 44-88)  Min: 5  Max: 90) |
| INR  (RV 0.8-1.3) | 1.18  (IQR: 1.1 – 1.29)  Min: 0,82  Max: 18,6 | 1.25  (IQR: 1.15-1.4)  Min: 0.82  Max: 18.8 |
| D-dimer  (RV: <500)  Units: ng/dL | 761  (IQR: 462.7 – 1369)  Min: 1.15  Max: 74625 | 1300  (IQR: 647.7-3231.7)  Min: 179  Max: 304000 |
| Creatine-kinase  (RV: 20-170)  Units: U/L | 80  (IQR: 47-154)  Min: 10  Max: 5634 | 89  (IQR: 53.2-179.7)  Min: 9  Max: 424000 |
| C-reactive protein  (RV: 1-5)  Units: mg/L | 65.9  (IQR: 23.4 – 122.1)  Min: 0.1  Max: 627.7 | 105  (IQR: 51.2 – 198.9)  Min: 0.3  Max: 672.3 |
| Procalcitonin  (RV: <0.5)  Units: ng/mL | 0.1  (IQR: 0.06-0.26)  Min: 0  Max: 55.02 | 0.13  (IQR: 0.06-0.43)  Min: 0.01  Max: 114 |
| Interleukine-6  (RV: <5.9)  Units: pg/mL |  | 27.8  (IQR: 12.4-61.8)  Min: 1.95  Max: 10000 |
| Ferritin  (RV: 15-150)  Units: ng/mL |  | 979  (IQR: 444.4-1819)  Min: 14.9  Max: 51805 |

*RV: Reference value. IQR: inter-quartile range. Min: Minimum. Max: Maximum. LDH: Lactate dehydrogenase. INR: international normalized ratio.*

Supplementary table 7: Frequency of abnormal values on admission and during the hospitalization period.

|  | % abnormal on admission | % abnormal during hospitalization | Adjusted p-value |
| --- | --- | --- | --- |
| Leukocytes  (RV 4-10)  Units: count x 10^9^/L | 167 / 574  (29.1%). | 390 / 572  (68.2%) | <0.001 |
| Lymphocytes (RV 0.9-5.2)  Units: count x 10^9^/L | 244 / 576  (42.4%) | 365 / 576  (63.4%) | <0.001 |
| Hemoglobin  (RV 12-16)  Units: count x 10^9^/L | 119 / 574  (20.7%) | 278 / 574  (48.4%) | <0.001 |
| Platelets  (RV 150-400)  Units: count x 10^9^/L | 147 / 557  (26.4%) | 268 / 554  (48.4%) | <0.001 |
| LDH  (RV 135-250)  Units: U/L | 362 / 563  (64.3%) | 451 / 573  (78.7%) | <0.001 |
| Glomerular filtration rate (corrected by body are)  (RV >90)  Units: ml/min/1.73m^3^ | 386 / 553  (69.8%)  *GFR 60-89: 232 (40.3%)*  *GFR 30-59: 112 (19.4%)*  *GFR 15-29: 34 (5.9%)*  *GFR <15: 7 (1.2%)* | 436 / 561  (77.7%)  *GFR 60-89: 221 (38.4%)*  *GFR 30-59: 130 (22.6%)*  *GFR 15-29: 58 (10.1%)*  *GFR<15: 26 (4.5%)* | <0.001 |
| INR  (RV 0.8-1.3) | 131 / 561  (23.4%) | 215 / 572  (37.6%) | <0.001 |
| D-dimer  (RV: <500)  Units: ng/dL | 390 / 544  (71.7%) | 487 / 572  (85.1%) | <0.001 |
| Creatine-kinase  (RV: 20-170)  Units: U/L | 49 / 349  (21.6%) | 132 / 500  (26.4%) | <0.001 |
| C-reactive protein  (RV: 1-5)  Units: mg/L | 520 / 564  (92.2%) | 553 / 573  (96.5%) | <0.001 |
| Procalcitonin  (RV: <0.5)  Units: ng/mL | 67 / 467  (14.3%) | 115 / 496  (20%) | <0.001 |
| Interleukine-6  (RV: <5.9)  Units: pg/mL |  | 429 / 480  (89.4%) |  |
| Ferritin  (RV: 15-150)  Units: ng/mL |  | 516 / 547  (94.3%) |  |

*RV: Reference value. LDH: Lactate dehydrogenase. GFR: Glomerular Filtration Rate. INR: international normalized ratio. P-value adjusted for multiple comparisons.*

Supplementary table 8. Results of the regression analysis in the association between chronic neurological disorders and abnormal laboratory parameters.

|  | OR | 95% CI | Adjusted p-value |
| --- | --- | --- | --- |
| Abnormal leukocyte count on admission (RV 4-10 x 10^9^/L) | 1.512 | 0.920-2.483 | 0.103 |
| Abnormal worst leukocyte count during hospitalization  (RV 4-10 x 10^9^/L) | 1.514 | 0.890-2.574 | 0.126 |
| Lymphopenia (<900 lymphocytes x 10^9^/L) on admission | 1.130 | 0.703-1.815 | 0.615 |
| Lymphopenia (<900 lymphocytes x 10^9^/L) during hospitalization | 1.152 | 0.691-1.920 | 0.587 |
| Anemia (<12^9^ units / L) on admission | 1.383 | 0.803-2.381 | 0.242 |
| Anemia (<12^9^ units / L) during hospitalization | 1.553 | 0.961-2.510 | 0.072 |
| Abnormal platelet count on admission  (RV 150-400) | 1.381 | 0.819-2.328 | 0.226 |
| Abnormal platelet count during hospitalization (RV 150-400) | 1.388 | 0.862-2.236 | 0.178 |
| Increased LDH on admission (>250 Units / L) | 1.345 | 0.811-2.231 | 0.251 |
| Increased LDH during hospitalization (>250 Units / L) | 1.183 | 0.634-2.207 | 0.597 |
| Abnormal GFR on admission (<90 ml/min/1.73m^3^) | 1.250 | 0.607-2.574 | 0.545 |
| Abnormal GFR during hospitalization (<90 ml/min/1.73m^3^) | 1.416 | 0.626-3.202 | 0.403 |
| Increased INR on admission (>1.3) | 1.140 | 0.650-1.999 | 0.646 |
| Increased INR during hospitalization (>1.3) | 1.859 | 1.147-3.011 | 0.012 |
| Abnormal D-dimer on admission (>500 ng/dL) | 1.201 | 0.660-2.187 | 0.549 |
| Abnormal D-dimer during hospitalization (>500 ng/dL) | 1.351 | 0.635-2.875 | 0.435 |
| Increased CPK on admission (>170 U/L) | 0.490 | 0.165-1.450 | 0.197 |
| Increased CPK during hospitalization (>170 U/L) | 1.348 | 0.768-2.365 | 0.298 |
| Increased CRP on admission (>5 mg/L) | 1.662 | 0.561-4.920 | 0.359 |
| Increased CRP during hospitalization (>5 mg/L) | 4.466 | 0.507-39.323 | 0.178 |
| Increased procalcitonin on admission (>0.5 ng/ml) | 1.204 | 0.604-2.399 | 0.599 |
| Increased procalcitonin during hospitalization (>0.5 ng/ml) | 1.845 | 1.079-3.155 | 0.025 |
| Increased interleukine-6 during hospitalization (>5.9 pg/ml) | 1.096 | 0.422-2.849 | 0.851 |
| Increased ferritin during hospitalization (>150 ng/ml) | 0.983 | 0.292-3.312 | 0.978 |

*OR: Odds ratio. CI: Confidence Interval. LDH: Lactate dehydrogenase. GFR: Glomerular Filtration Rate. INR: international normalized ratio. P-value by age, modified Rankin scale and sex.*
